# EV-associated HOTAIR in cancer cell communication: a distance-aware review of functional transfer claims

**DOI:** 10.3389/fcell.2026.1891799

**Published:** 2026-08-10

**Authors:** Xitan Wang, Han Li

**Affiliations:** Department of Oncology, Zibo Central Hospital, Zibo, Shandong, China

**Keywords:** cancer cell communication, causal continuity, circulating RNA, extracellular vesicles, functional transfer, HOX transcript antisense intergenic RNA, long non-coding RNA

## Abstract

HOX transcript antisense intergenic RNA (HOTAIR) is classically definedas an intracellular long non-coding RNA that scaffolds PRC2/LSD1 to remodel chromatin and functions as a competing endogenous RNA. Its detection in extracellular vesicles (EVs) and circulating biofluids has extended this view towards intercellular and potentially systemic roles in cancer. However, EV-associated RNA claims often combine evidence obtained at different biological scales, making it difficult to distinguish functional cell-to-cell transfer from circulating detection. Here, we review the HOTAIR literature through a distance-aware lens—a descriptive interpretive aid rather than an established evidence taxonomy—that separates intracellular activity (D0), paracrine or microenvironmental transfer (D1), and systemic circulation (D2). At each scale, we ask whether the receiving cell or tissue has been functionally validated. Within selected D0–D1 contexts, EV-associated HOTAIR has been implicated in local recipient-cell reprogramming, including effects on B cells, macrophages, endothelial cells, and stromal or tumour cells through metabolic, transcriptional, and signalling routes. Several mechanistically distinct pathways recur on checkpoint-associated and immunosuppressive endpoints, particularly PD-L1-linked phenotypes; we describe this as a recurrent checkpoint-associated convergence pattern rather than as a new biological paradigm. Circulating HOTAIR remains a valuable discovery and monitoring signal; however, claims that it functions as a distal messenger should be qualified unless downstream recipient-cell function, cytosolic delivery and causal continuity are demonstrated. Circulation-scale studies can motivate productive paracrine- and intracellular-scale follow-ups, even when distal function is not established. We describe the mismatch between abundant detection and insufficiently demonstrated functional continuity as an evidential trough. This review therefore positions EV-associated HOTAIR as a useful case for matching cancer cell communication claims to the scale and quality of evidence that supports them while preserving the biomarker value of circulating RNA studies.

## 1 Introduction: why cancer cell communication claims need to be read by scale

A central challenge in cancer extracellular-RNA biology is distinguishing molecular detection from functional communication. Tumour, stromal, endothelial, and immune cells constantly exchange extracellular vesicles (EVs), soluble factors, and contact-dependent signals; however, the claim that EV-associated RNA functions as a transferable signal requires more than its release or detection. It requires, rather, a defined source, a biologically plausible cargo, recipient-cell uptake, evidence of intracellular delivery or action, and a measurable recipient phenotype. Systemic hormones meet this standard through defined receptors and dose-response relationships. Extracellular RNA often lacks an equivalent receptor framework, so the same language of directed communication can rest on a less complete chain of proof. That asymmetry is the problem this review addresses.

Three features make EV-associated RNA unusually easy to overread as cancer cell communication. The first is no defined receptor: an RNA in plasma has no receptor framework comparable to a hormone, so “it is present and the phenotype changed” is too readily read as “it caused the phenotype.” The second is sub-stoichiometric payloads: quantitative research indicates that in standard preparations, the average copy number of a given small RNA is far below one molecule per vesicle (Chevillet et al., 2014), and the cognate argonaute proteins are themselves hard to detect in common EV isolates (Wang H. et al., 2022). The third feature is that release is not delivery: even when a vesicle is taken up, whether its RNA escapes the endosome into the cytosol to act is contested (Kanada et al., 2015). None of this makes EV-RNA signalling impossible. Broader EV literature, including a recent preprint review, has discussed how exosomal cargoes such as non-coding RNAs and chromatin-regulatory components may participate in epigenetic rewiring and therapy resistance in cancer (Verma et al., 2026). However, for HOX transcript antisense intergenic RNA (HOTAIR) specifically, functional transfer language should be reserved for studies that directly examine EV-associated HOTAIR, recipient cell responses, and causal continuity. This is why EV-HOTAIR claims must be read against the scale at which the evidence was obtained, which is what the rest of this review does.

### 1.1 Scope and literature search

This review was designed as a narrative, concept-driven review rather than a systematic review or meta-analysis. We searched PubMed, Web of Science, and Scopus from database inception to June 2026, using combinations of “HOTAIR,” “lncRNA HOTAIR,” “extracellular vesicle(s),” “exosome(s),” “EV-HOTAIR,” “circulating HOTAIR,” “functional transfer,” “intercellular communication,” “tumour microenvironment,” “immune escape,” “PD-L1,” “macrophage,” “B cell,” “endothelial cell,” “angiogenesis,” “biomarker,” and “cancer.” Reference lists of retrieved articles and relevant EV-methodology position papers were also screened. English-language primary studies were prioritised when they addressed intracellular HOTAIR mechanisms, EV-associated or circulating HOTAIR, recipient-cell phenotypes, or methodological issues relevant to EV-RNA interpretation; reviews and position papers were used for background and standards. Because the purpose was interpretive rather than quantitative, studies were selected for relevance to the scale-of-evidence question rather than by predefined meta-analytical criteria. The final cited set includes the primary EV-HOTAIR transfer studies and the circulating HOTAIR biomarker/resistance studies discussed in Section 6. The limited number of functionally validated transfer studies is itself relevant to the review’s central argument and is noted where the corresponding claims are made.

## 2 From intracellular HOTAIR biology to a scale-aware reading of cell communication

### 2.1 Intracellular baseline (distance 0)

HOTAIR is transcribed from the HOXC locus and acts intracellularly in two well-supported ways. It is a modular scaffold in the nucleus: its 5′ region binds PRC2 (through EZH2) to deposit repressive H3K27me3, while its 3′ region engages the LSD1/CoREST/REST complex to strip activating H3K4me2, jointly silencing target loci, including the posterior HOXD cluster (Rinn et al., 2007; Tsai et al., 2010). It acts as a ceRNA in the cytoplasm: sponging miR-301a-3p to de-repress FOSL1 (Guo et al., 2022), miR-218 to modulate PDE7A (Wei et al., 2020), and working through miR-125/miR-214 axes on HK2 and the β-catenin/MGMT pathway in temozolomide resistance (Zhang et al., 2020; Lan et al., 2024). This is the nearest communication scale (D0): short causal chain and highest certainty. This is the calibration baseline, not the subject of this review.

### 2.2 Interpretive challenge in EV-associated HOTAIR studies

The field has increasingly moved from asking what HOTAIR does inside a single cancer cell to asking whether it is packaged into EVs and participates in communication between cancer cells and their surrounding microenvironment. HOTAIR has been reported in exosomes and circulating biofluids, including serum and circulating exosomal fractions, and has been correlated with diagnostic or prognostic features in several cancers (Wang et al., 2014); selected studies also report donor EV-HOTAIR uptake and recipient-cell reshaping (Xie et al., 2023; Wang J. et al., 2022; Lu et al., 2025). The recurring interpretive risk is a single move: describing “detectable circulating RNA” in the language of a directed endocrine-like messenger. We respond not by dismissing the topic but by matching each claim to the scale and quality of evidence that supports it.

### 2.3 The lens, stated plainly first

In one sentence before any terminology: we match each communication claim to the biological scale at which its evidence was obtained and ask whether the receiving cell or tissue has actually been shown to respond functionally. Operationally, this gives three stable descriptive distance categories within the literature: D0 intracellular activity, D1 paracrine or microenvironmental transfer, and D2 systemic circulation. These labels are not an established evidence taxonomy and do not indicate increasing evidential strength. They are used as a practical reading aid: greater biological distance generally entails more dilution, more barriers, and more opportunities for confounding, which is why the evidentiary bar rises when claims move from local transfer to distal messaging.

#### 2.3.1 Where the evidence breaks

**FIGURE 1:**
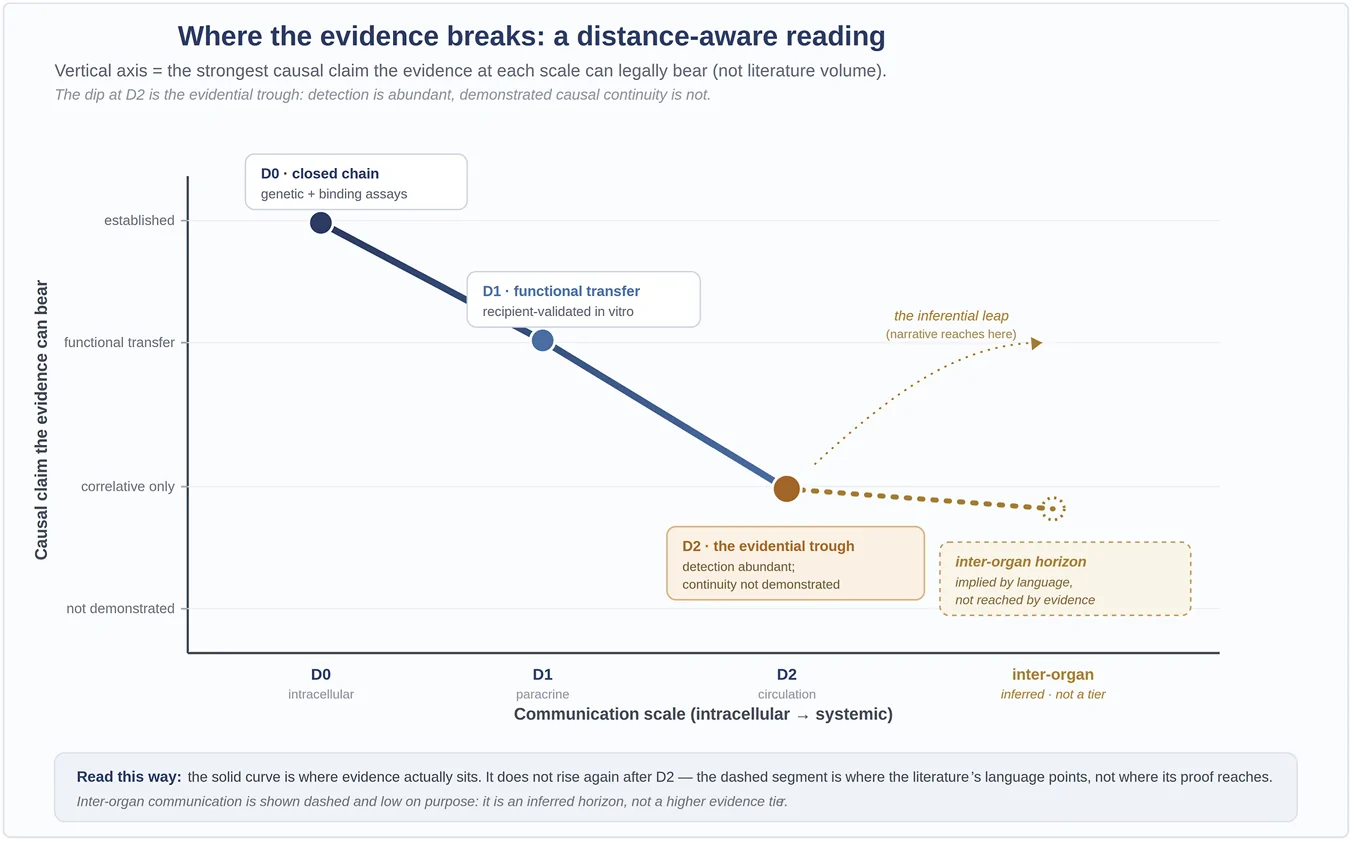
Distance-aware evidence lens for extracellular-vesicle (EV)-associated HOTAIR claims. Extracellular HOTAIR evidence is organised according to the communication scale at which it is obtained and the degree of demonstrated causal continuity. Intracellular HOTAIR activity represents D0, where molecular binding and perturbation studies provide the strongest causal support. Paracrine or microenvironmental EV transfer represents D1, where recipient-cell functional changes have been shown in several local contexts. Circulating HOTAIR represents D2, where biofluid detection and biomarker associations are abundant but downstream recipient identity, cytosolic release and functional continuity are usually not demonstrated. The gap between detection-rich circulation studies and language implying distal functional communication is termed the evidential trough. Inter-organ communication is shown as an unresolved horizon rather than an established HOTAIR category, indicating a biologically plausible but currently unclosed causal chain.

Across these scales, the pattern is not “farther is uniformly weaker.” D0 is relatively closed; selected D1 studies provide recipient-validated functional transfer; then, at the circulation layer, detection becomes abundant while demonstrated function does not follow. We call this gap the “evidential trough”. Its definition is deliberately precise and is the hinge of this review: *the trough does not arise because circulation-scale studies are intrinsically weak but because some detection-level observations are interpreted in ways that imply distal functional communication without demonstrating contiguous causal continuity.* The distinction we draw is therefore not between strong and weak studies but between what circulation-scale evidence shows and what some statements about it imply—an inferential step we examine below in “Discussion” rather than treat it as an established tier. This scale-dependent evidence pattern is summarised in Figure 1 and operationalised in Box 1.

Box 1. **Distance-aware interpretative categories for EV-associated HOTAIR claims.**

*D0 intracellular/autocrine*. HOTAIR acts within its cell of origin. Evidence concerns intracellular HOTAIR function supported by perturbation, molecular binding, chromatin, ceRNA, or signalling assays.

*D1 paracrine/microenvironment*. The evidence concerns EV-associated HOTAIR moving between nearby cells within the tumour microenvironment, with recipient-cell uptake and phenotypic reprogramming tested. D1 is the furthest scale at which selected HOTAIR studies currently provide recipient-cell functional read-outs, although cytosolic release and donor-specific rescue remain unevenly reported.

*D2 systemic circulation*. The evidence concerns HOTAIR detected in plasma, serum, or EV-enriched biofluids. This layer is valuable for biomarker discovery, monitoring, and hypothesis generation, but it does not by itself establish distal functional communication.

*Evidential trough* denotes the gap between detection-rich D2 studies and claims that imply recipient-cell function or distal messenger activity without demonstrated source-to-recipient causal continuity.

*Important clarification*. D0–D2 are author-proposed, distance-based interpretative categories. They are not established EV evidence types, not a formal grading system, and not a claim that evidential strength necessarily increases or decreases with biological distance. Inter-organ communication is treated separately as an unresolved horizon rather than as a fourth distance category.

### 2.4 The central question and our position

This produces the central question for EV-HOTAIR biology: when is vesicle-associated HOTAIR a functional cargo that reprograms a recipient cell and when is it a circulating by-product or biomarker of a cellular state? Section 1 gave the quantitative reason why this is a genuine question rather than idle doubt: sub-stoichiometric payloads, scarce Argonaute, and contested cytosolic escape (Chevillet et al., 2014; Kanada et al., 2015; Albanese et al., 2021). Multiple independent analyses keep questioning extracellular RNA’s function, not merely its detectability (Mateescu et al., 2017; Turchinovich et al., 2011). We answer it by a simple rule: only evidence at a scale with recipient-cell functional support may use “functional transfer” language; circulation-scale detection, however valuable for discovery and monitoring, may not.

To keep the lens operational rather than merely narrative, Table 1 summarises this distance-based interpretation of EV-associated HOTAIR claims, while Box 2 and Table 2 (Section 5.4) set out the minimum controls that would support functional, rather than associative, D1 transfer language. For D2 studies, the relevant standard is different: circulating detection can support discovery, monitoring, and hypothesis generation, but distal messenger language requires downstream recipient identity, cytosolic delivery, and causal continuity. This distinction is why the inter-organ horizon is discussed as a falsifiable program rather than as an established category of HOTAIR biology.

**TABLE 1:** Distance-based interpretation of EV-associated HOTAIR claims and minimum evidence required.

Distance layer	What is being shown	Claim reasonably supported	Main limitation	Minimum evidence required
D0: intracellular/autocrine	HOTAIR function within the same cell	Intracellular HOTAIR activity may be stated when perturbation, binding, or pathway data support it	Does not establish EV-mediated transfer	Perturbation/rescue, ChIRP/RIP/CLIP, or equivalent binding assays, pathway read-outs
D1: paracrine/microenvironment	Local EV-associated transfer with recipient-cell phenotype	Local recipient-cell reprogramming may be claimed when EV dependence and HOTAIR specificity are shown	Uptake may not equal cytosolic delivery; co-transferred cargo may confound attribution	MISEV-aligned EV validation, EV-depletion/mock controls, RNase ± detergent, donor HOTAIR KD/KO, rescue, uptake plus delivery read-out, recipient phenotype
D2: systemic circulation	HOTAIR detection in serum, plasma, or EV-enriched biofluids	Discovery, diagnosis, prognosis, and monitoring; hypothesis generation for D1/D0 follow-up	Does not establish distal messenger function or recipient-cell causality	Carrier definition, isolation method, particle/protein markers, spike-in and normaliser justification, haemolysis/sample handling, independent validation
Inter-organ horizon	Putative organ-to-organ EV-HOTAIR communication	Hypothesis or falsifiable program only	Not established for HOTAIR	Tissue-specific EV tracing, donor-organ perturbation, physiological-dose cargo quantification, recipient-organ phenotype rescue

D0–D2 denote biological distance, not a formal hierarchy of evidential strength. The inter-organ row is shown as an unresolved horizon rather than as an established HOTAIR category. The evidential trough sits at D2 when abundant detection is interpreted as distal functional communication without demonstrated continuity.

Box 2. **Minimum evidence for a paracrine (D1) transfer claim.**

1. EV-depletion or release-blockade controls: Removal or inhibition of EVs should abolish the recipient-cell phenotype.

2. Cargo-protection controls: The HOTAIR signal should resist RNase treatment unless detergent is added, supporting vesicular protection of the RNA cargo.

3. Donor-cell loss-of-function with rescue: HOTAIR depletion in donor cells should remove the recipient-cell effect, and HOTAIR re-expression should restore it.

4. Recipient-cell delivery read-out: Internalized HOTAIR should be shown to reach a functional intracellular compartment, because uptake alone does not establish cytosolic delivery.

5. A defined and quantified recipient endpoint: The recipient phenotype should ideally demonstrate donor dependence or dose dependence.

**TABLE 2:** Reporting features of representative EV-HOTAIR transfer studies, read against MISEV2023 (Welsh et al., 2024).

Study; model/EV source	Isolation	Markers and sizing	RNase protection	Donor HOTAIR control	Recipient delivery and endpoint
Xie et al. (2023), BBRC; CRC MC38 exo → B cells; plasma	Differential UC (120,000 g × 2)	TEM, NTA; no EV tetraspanin/neg-marker blot (CD63/81/9 via TCGA only)	✓ (RNase A ± Triton)	Donor KD + OE; GW4869 block, *in vivo*	PD-L1 induction (dose/time); CD8 suppression; *in vivo*
Wang J. et al. (2022), BMC cancer; LSCC exo → macrophages	Conditioned medium (not detailed)	TEM (30–150 nm), NTA; CD9/81/63; no neg-marker	—	Donor si-HOTAIR	M2 polarisation (CD163/206); EMT, metastasis
Lu et al. (2025), Chinese†; CAF exo → ESCC	CAF/NF conditioned medium (full text not reviewed)	Not specified (abstract)	—	KD in recipient, not donor; CAF- vs. NF-exo	Proliferation/migration/invasion; EMT; glycolysis
Tuersong et al. (2025), J cell commun signal; EC EVs → resistant EC, T cells	UC + 0.22 µm filter (100,000 g × 2)	TEM, NTA; CD63/81/TSG101; calnexin neg	—	Donor-EV si-HOTAIR; OE-CDH2 rescue	Dil uptake; PTX resistance, PD-L1, T-cell cytotoxicity
Ma et al. (2017), Am J Transl res; glioma CM → endothelial	None—CM used directly; EVs not isolated	None (no TEM/NTA/blot)	✓ (CM, RNase + Triton)	Donor si-HOTAIR; VEGFA-OE rescue	Endothelial proliferation/migration/tube formation
Zhang et al. (2022), Int J Nanomedicine‡; endometriosis ESC exo → ESC/HUVEC	Sequential UC (100,000 g × 2)	TEM, NTA; CD9/63; no neg-marker/TSG101	—	Donor-exo si-/OE-HOTAIR; miR-761/si-HDAC1 rescue	Dil uptake; tube formation; *in vivo*
Born et al. (2022), Adv Healthc Mater‡; engineered MSC-EV → endothelial/wound	Differential UC + 300 kDa wash	TEM, NTA; CD63/TSG101; calnexin and albumin neg; VEGFA screen	— (loading by qPCR/gel)	Engineered OE only; ctrl-transfected MSC	Membrane-label uptake; recipient HOTAIR ↑∼21×, VEGFA ↑∼11×; *in vivo*
Wang X. et al. (2022), Cell Death Dis; serum EVs (GBM) → GBM cells	From serum (equal volume)	NTA; CD63/9/TSG101; no neg-marker	—	Serum-derived; recipient HOTAIR raised (no donor KO/rescue)	Co-culture → proliferation/invasion/TMZ resistance; *in vivo*

UC, ultracentrifugation; ✓, reported; —, not reported.

^†^ Information available from abstract.

^‡^ Non-cancer or therapeutic model cited as mechanistic context. This table is intended to bound interpretation rather than to invalidate individual studies.

## 3 The biogenesis checkpoint: is HOTAIR selectively loaded into EVs?

Before investigating what extracellular HOTAIR does, we ask how it got out, and whether that exit is selective. The answer sets how far the circulation-layer reading can go. If loading is not selective, circulating HOTAIR is more plausibly leakage than cargo.

### 3.1 Sorting proteins and putative motifs

Selective RNA loading into EVs, best established for miRNAs and increasingly discussed for other ncRNA classes, is thought to involve sequence- or structure-specific RNA-binding proteins. The repeatedly implicated candidates include hnRNPA2B1, YBX1, and SYNCRIP/hnRNPQ, each proposed to recognise short “EXOmotifs” or RNA features and chaperone transcripts towards EV-associated compartments (Villarroya-Beltri et al., 2013; Sh et al., 2016; Shurtleff et al., 2017; Santangelo et al., 2016). Whether HOTAIR itself carries a defined EXOmotif or structured docking element that licenses preferential export has not been conclusively mapped. A defined, saturable motif would argue for regulated secretion; its absence favours stochastic incorporation.

### 3.2 Why the sorting question matters here

The field has not converged on a consistent motif–protein–transcript code; recent expert appraisals note a lack of agreement on the relevant motifs, RNA-binding proteins, and preferentially sorted RNAs across studies and cell types (Mateescu et al., 2017; Liu and Halushka, 2025). We therefore make no determinate claim about how HOTAIR is loaded. The consequence is concrete: without demonstrated selective packaging, detecting HOTAIR in circulation cannot by itself indicate a purpose-built axis, and its default reading remains a correlative liquid snapshot. The biogenesis step is the first gate—an unproven node drawn explicitly rather than assumed away.

## 4 Recipient-cell reprogramming and checkpoint convergence (within D0-D1)

Crossing from inside the cell (D0) into the adjacent microenvironment (D1), HOTAIR shows real paracrine capacity. For a cell-biological reading, the most useful approach is not a catalogue of immune effects cell-by-cell but a cell-communication pattern: EV-associated HOTAIR can alter recipient-cell state through metabolic, transcriptional, and signalling routes, with several routes converging on checkpoint-associated phenotypes. Section 4.3 frames that pattern as recurrence rather than declared specificity.

The recipient cells discussed below sit within the tumour immune microenvironment, where extracellular vesicles are an established route by which tumours reshape immunity; tumour-derived EVs deliver immunosuppressive cargo—proteins such as PD-L1, Fas-L, and TGF-β along with a range of regulatory RNAs—to T cells, natural killer cells, macrophages, dendritic cells, and myeloid-derived suppressor cells, biasing the microenvironment towards tolerance (Liu et al., 2025). EV-associated PD-L1 is itself a well-described axis, with vesicle-borne PD-L1 suppressing T-cell activity in local and systemic immune contexts and contributing to checkpoint resistance (Lu and Yang, 2024). Against this backdrop, HOTAIR is best read not as a unique immune effector but as one candidate RNA cargo amongst many that converge on shared immunosuppressive endpoints. The examples that follow—regulatory, PD-L1-high B cells (Xie et al., 2023), M2-polarised macrophages (Wang J. et al., 2022), intracellular HOTAIR-associated NF-κB immune escape (Wang et al., 2021), and an EV-HOTAIR axis that raises PD-L1 and blunts T-cell cytotoxicity (Tuersong et al., 2025)—can therefore be read against this broader pattern, with the scale-of-evidence caveats of Section 2 applying to each.

### 4.1 One concrete cascade, in full

We consider the clearest example before any abstraction. In colorectal cancer, tumour-derived exosomal HOTAIR is taken up by infiltrating B cells (Xie et al., 2023). In the recipient cytosol, it binds the glycolytic rate-limiting enzyme PKM2 and physically occludes the site engaged by PKM2’s cognate E3 ubiquitin ligase, sparing PKM2 from proteasomal degradation (Xie et al., 2023). The resulting PKM2 accumulation, acting in its kinase-competent form, drives STAT3 phosphorylation, which transactivates PD-L1 (Xie et al., 2023). This gives the B cell a regulatory (Breg) phenotype that, through PD-L1/PD-1, reciprocally disables CD8 (+) T-cell cytotoxicity (Xie et al., 2023): one molecule, one contiguous chain, from inside the recipient cell to a B-cell→T-cell synapse effect, entirely within the D0–D1 range.

### 4.2 Three more routes, same endpoint

The same endpoint is reached by routes that share no proximal cause. For tumour-intrinsic, UBXN1/NF-κB, HOTAIR in glioma lowers UBXN1 (a negative regulator upstream of NF-κB), relieving restraint on IκBα/NF-κB phosphorylation and nuclear translocation, thus driving tumour PD-L1 and immune escape (Wang et al., 2021). For tumour→T cell, miR-375/CDH2 EV-delivered HOTAIR in paclitaxel-resistant oesophageal carcinoma raises PD-L1 via a miR-375/CDH2 axis, thus blunting T-cell cytotoxicity (Tuersong et al., 2025). For tumour→macrophage, PI3K/AKT, exosomal HOTAIR pushes macrophages towards the immunosuppressive M2 state via PI3K/p-AKT/AKT, thus promoting laryngeal-carcinoma progression (Wang J. et al., 2022).

### 4.3 Recurrence, not declared specificity

PD-L1 is a generic immune-evasion endpoint, so HOTAIR convergence should not be overstated as strict specificity. Such a claim would require systematic comparison against other microenvironmental factors and exclusion of IFNγ-, TGFβ-, or stress-driven checkpoint convergence—comparisons not performed here. The defensible claim is therefore about recurrence across selected studies, not exclusivity: observations span partially independent routes (PKM2/STAT3, UBXN1/NF-κB, miR-375/CDH2 and PI3K/AKT) and several compartments (tumour cells, B cells and macrophages), yet they recur on checkpoint-associated or immunosuppressive endpoints. We describe this cautiously as a recurrent checkpoint-associated convergence pattern across selected HOTAIR studies rather than as a distinct HOTAIR-specific immune program or a new biological paradigm.

### 4.4 Three modes inside the convergence

**FIGURE 2:**
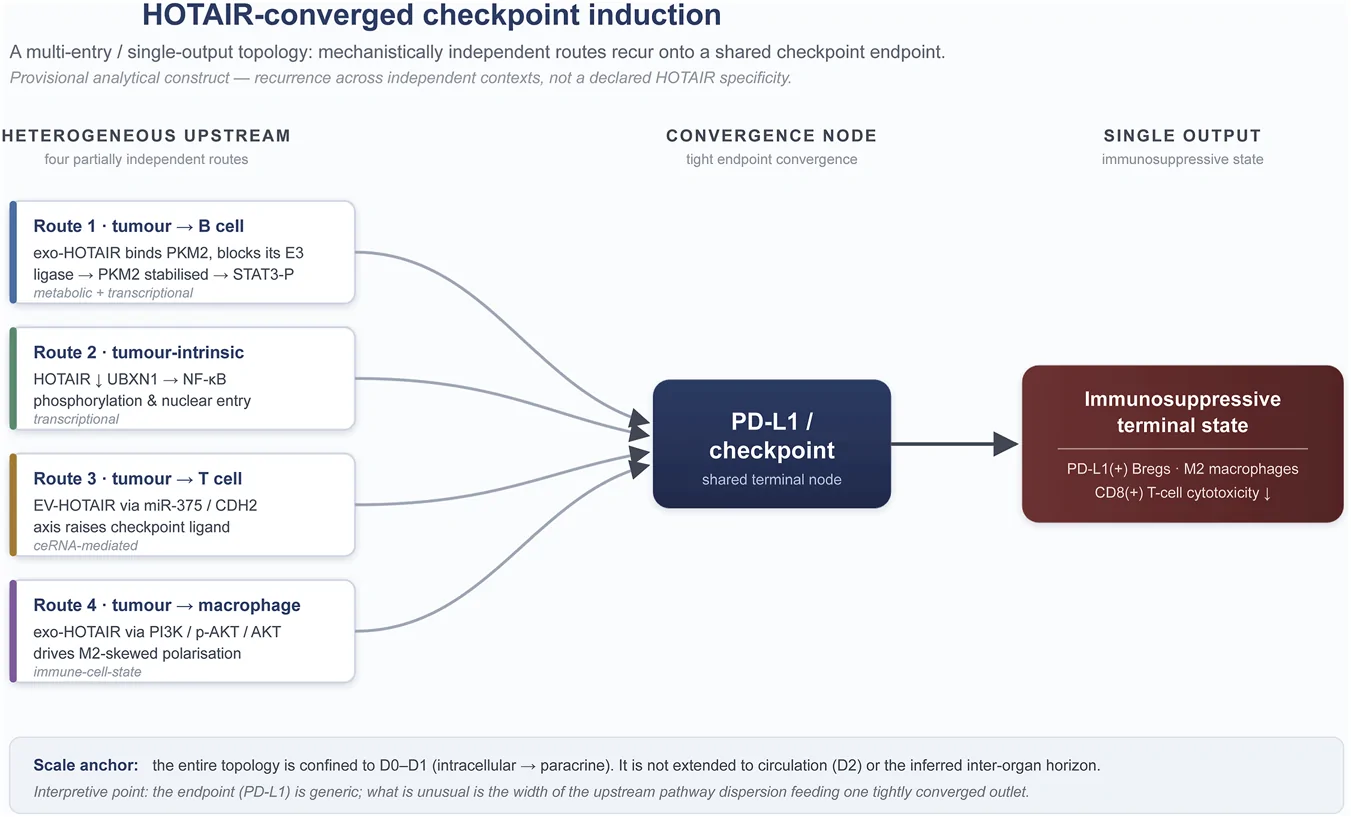
HOTAIR-associated checkpoint convergence across mechanistically distinct routes. Within selected D0–D1 contexts, HOTAIR-related pathways converge on checkpoint-associated immunosuppressive outputs through multiple upstream mechanisms. Tumour-derived exosomal HOTAIR can stabilise PKM2 in recipient B cells, promoting STAT3 phosphorylation and PD-L1-associated Breg activity; tumour-intrinsic HOTAIR can relieve UBXN1-mediated restraint on NF-κB signalling; EV-delivered HOTAIR can act through a miR-375/CDH2 axis to increase PD-L1 and impair T-cell cytotoxicity; exosomal HOTAIR can participate in PI3K/AKT-linked macrophage M2 polarisation. The figure summarises this as a recurrent convergence pattern rather than a claim of strict HOTAIR specificity, since PD-L1 and immune-suppressive states are shared endpoints for many tumour-associated pathways.

Structurally, the routes converge in three modes: a transcriptional mode (STAT3 and NF-κB independently transactivating PD-L1), a metabolic mode (PKM2 stabilisation coupling a Warburg-type node to checkpoint output), and an immune-cell-state mode (the readout is a stabilised suppressive identity—M2, Breg). For the same destination is three non-overlapping channels, which is what makes “convergence” a structural observation rather than a coincidence. This multi-entry, checkpoint-associated convergence pattern is summarised in Figure 2.

### 4.5 Why HOTAIR is a permissive substrate

Why would one lncRNA recur across such independent contexts? Not from a formal topological property but from its multi-domain interactome capacity as a long RNA: the same molecule can be a protein scaffold (PRC2/LSD1), a miRNA sponge, a metabolic-enzyme stabiliser (PKM2) and an EV-transmissible cargo, operating in parallel across chromatin, ceRNA buffering, metabolism, and intercellular transfer. This versatility—a summary of known biochemistry, not a proven sorting mechanism—makes heterogeneous inputs capable of a convergent output. Because the sorting machinery itself is contested (Section 3.2), we claim only that functional pluripotency makes the recurrence plausible and not mechanistically inevitable.

### 4.6 Boundaries: adaptive, innate, and CNS

The B-cell route is the HOTAIR-characteristic adaptive node (tumour→EV→Breg→CD8 (+) T suppression) (Xie et al., 2023), anchored at D1. On the innate side, EV-HOTAIR is proposed to participate (not “drive”) in M2 reprogramming via PI3K/p-AKT/AKT (Wang J. et al., 2022), since evidence is largely *in vitro* co-culture without *in vivo* donor-specific reversion. In CNS injury, demyelination, and Parkinsonian models, HOTAIR is reported in microglial activation and neuroinflammation (Cheng et al., 2021; Wang Y. et al., 2022; Duan et al., 2018). The boundary that must be stated is that the evidence supports HOTAIR as a secondary correlate of peripheral-to-central inflammation or an *in vitro* uptake phenomenon, not a direct driver crossing the blood–brain barrier. CNS material is confined to associative statements—a pre-emptive closure of the “crosses or merely correlates” question.

## 5 Cross-cell functional relay in the tumour microenvironment (distance 1)

This layer represents the furthest scale at which functionally validated, contiguous transfer is currently demonstrated in the HOTAIR literature—strictly preceding the evidential trough of systemic circulation. There is one up-front boundary: even when a recipient phenotype changes in co-culture, it remains unresolved whether EV cargo truly escapes endosomes into the cytosol (Somiya, 2020). This evidence is therefore “*in vitro* support for functional transfer,” not “proof of delivery,” and wording is constrained accordingly.

### 5.1 Stroma–tumour relay

A recent study in oesophageal squamous-cell carcinoma reported that CAF-derived exosomal HOTAIR was associated with enhanced tumour-cell proliferation, migration, invasion, EMT, and glycolytic features, including HK2, ECAR, and ATP/ADP changes (Lu et al., 2025). Because the available reporting of EV characterisation and donor-specific controls is limited, we treat this study as supportive but not definitive evidence for a D1 stroma–tumour metabolic relay.

### 5.2 Tumour–endothelial relay

Glioma cells induce VEGFA and deliver HOTAIR via EVs to endothelium, thus promoting angiogenesis (Ma et al., 2017). Hypoxic papillary thyroid carcinoma raises exosomal miR-181a through HOTAIR/RELA to suppress GATA6 (Lu et al., 2024). In endometriosis, exosomal HOTAIR promotes angiogenesis via miR-761/HDAC1 and STAT3-linked inflammation (Zhang et al., 2022). Together, there is a lesion→endothelium pro-angiogenic relay at D1.

### 5.3 Context-dependent duality

EV-HOTAIR is not uniformly oncogenic: MSCs releasing HOTAIR-rich exosomes promote angiogenesis and wound-healing in diabetic mice (Born et al., 2022), while BM-MSC exosomes that downregulate HOTAIR/upregulate miR-221 ameliorate cerebellar degeneration in Parkinsonian rats (Shalaby et al., 2025). Any functional reading must be bound to donor cell type and recipient context rather than be asserted context-free.

### 5.4 Minimum evidence and reporting standards for EV-mediated transfer

The central question is whether an EV-associated transcript reaches and acts in a recipient cell; accordingly, the strength of any paracrine (D1) claim depends directly on how the underlying vesicles were isolated, characterised, and controlled. We read representative EV-HOTAIR transfer studies against the reporting framework of MISEV2023 (Welsh et al., 2024), including EV source, isolation strategy, positive and negative markers, sizing or imaging, cargo localisation, and transfer-specific controls. As summarised in Table 2, reporting across these studies is uneven rather than uniformly weak. Most studies include some combination of tetraspanin markers CD9, CD63, or CD81. electron microscopy, or nanoparticle-tracking analysis, whereas cargo-protection assays, negative or contaminant markers, donor-specific rescue, and cytosolic-delivery read-outs are less consistently reported. In several studies, donor-cell HOTAIR perturbation supports a transfer-associated phenotype, but cargo specificity is not always fully isolated from co-transferred EV components. This unevenness does not invalidate the studies—it defines how strongly the aggregate literature can support functional HOTAIR-transfer language and helps explain why detection-rich D2 studies should not be interpreted as completed distal communication chains.

We regard the following as minimal controls that license functional rather than associative language at this scale: EV-depletion or release-blockade controls showing that removal or inhibition of EVs abolishes the recipient phenotype; cargo-protection controls showing that the HOTAIR signal resists RNase unless detergent is added; donor cell loss-of-function with rescue, showing that HOTAIR depletion removes the recipient effect and re-expression restores it; a delivery read-out indicating that internalised HOTAIR reaches a functional compartment, since uptake is not the same as cytosolic delivery (Kanada et al., 2015; Somiya, 2020); a defined, quantified recipient endpoint, ideally with donor- or dose-dependence. Where a study omits donor-cell rescue or cargo protection, its transfer language should be read as associative rather than demonstrated.

#### 5.4.1 A quantitative plausibility check

A simple stoichiometric argument sharpens this point. Quantitative analyses of EV RNA indicate that, in unmanipulated preparations, a given small RNA is present at well below one copy per vesicle—often in the order of one molecule per hundred vesicles or fewer—and the Argonaute proteins required for canonical miRNA activity are scarce in common EV isolates (Chevillet et al., 2014; Wang H. et al., 2022). For a long, comparatively low-abundance transcript such as HOTAIR, the expected per-vesicle copy number is therefore unlikely to exceed 1 without selective loading or experimental enrichment. Internalised cargo must also escape the endosome to act, and this step is inefficient (Kanada et al., 2015). A recipient cell would therefore need to take up a large number of vesicles to accumulate even a few cytosolic copies. These considerations do not exclude EV-HOTAIR signalling, but they require quantitative caution: an endogenous D1 transfer claim becomes more plausible when cargo enrichment, recipient-cell copy number, dose-response behaviour, cytosolic availability, or target engagement is measured rather than assumed. They also help explain the contrast visible in Table 2—engineered, HOTAIR-overexpressing vesicles (Born et al., 2022) provide clearer evidence of measurable recipient delivery, whereas studies relying on unenriched preparations usually require additional controls before functional delivery can be inferred.

## 6 Circulating HOTAIR as a biomarker layer: discovery, monitoring, and the limits of cell communication claims (distance 2)

### 6.1 What circulating HOTAIR is good for

Before the critique, we consider the constructive point. Circulating-RNA studies remain indispensable for cell and cancer biology: they identify candidate communication axes and enable minimally invasive disease monitoring and surface biology worth pursuing—value that does not depend on resolving causal continuity. Circulating HOTAIR behaves as a systemic transcriptional shadow of tumour mass and, used as such, is genuinely useful. The argument here is not against this literature; it is about the language some studies adopt. This separation between detection and demonstrated transfer is not unique to cancer: in other tissue contexts, EV-associated RNAs are routinely detected and used to support minimally invasive biomarker development without proof of functional transfer (Verma et al., 2025a).

### 6.2 Where the reading breaks, and a technical attribution for the conflict

The circulation layer contains the overt conflict we report rather than smoothing over. Some studies are strongly positive—one reports a HOTAIR AUC (0.791) above CEA (0.737) (Li et al., 2017), and another reports near-perfect performance in thyroid cancer (Mahmoud et al., 2023), while a larger NSCLC cohort states that HOTAIR’s accuracy is no better than conventional markers and below NSE/CEA/CYFRA21-1 (Yao et al., 2022). Much of this conflict has concrete technical roots that are not always made explicit. (a) Isolation-method confounding: studies reporting high diagnostic value often use polymer-precipitation kits (ExoQuick-type) that co-precipitate large amounts of non-EV circulating ribonucleoprotein complexes; differential ultracentrifugation or size-exclusion chromatography can give different results, because the “EV-HOTAIR” measured is not operationally the same analyte across studies (Welsh et al., 2024; Murillo et al., 2019). (b) The reference-gene problem: quantifying circulating HOTAIR by qPCR needs a stable normaliser, yet GAPDH and U6 are unstable and matrix-dependent across biofluids—U6 in particular is poorly retained and variably degraded in plasma/serum—and normaliser choice alone can move reported fold-changes enough to flip a study’s conclusion (Chen et al., 2013; Gouin et al., 2017; Iempridee et al., 2018).

These technical axes—not biology alone—plausibly generate much of the conflict, and their existence is itself the argument: circulation-scale evidence is correlative, so a statement implying functional cell-to-cell or distal communication reaches past what this evidence shows. This is the evidential trough as defined in Section 2.3—not weak data, but interpretation reaching past demonstrated continuity. Framing these studies precisely does not diminish their value: the same circulating signals that are only correlative at this scale are frequently what motivate the controlled paracrine- and intracellular-scale experiments that can establish function.

#### 6.2.1 Reporting checklist for circulating-HOTAIR (D2) studies

For HOTAIR measured in serum, plasma, or biofluid vesicles, studies should report at least five items before diagnostic or monitoring claims are considered interpretable: pre-analytical handling, including specimen type, processing time, storage, freeze–thaw cycles, and haemolysis assessment; EV or carrier definition, including isolation method, particle/protein characterisation and whether the measured RNA is vesicle-associated, protein-bound, lipoprotein-associated or total extracellular RNA; RNase ± detergent controls to distinguish vesicle-protected HOTAIR from externally associated RNA; spike-in controls and empirically justified normalisers rather than assumed housekeepers such as U6 or GAPDH; analytical performance, including limit of detection, dynamic range, reproducibility, cohort size, and pre-specified cut-offs. These items concern interpretability, not novelty: a circulating-HOTAIR signal can remain clinically useful even when it does not establish cell-to-cell communication.

A high-profile illustration reinforces this reading: a frequently cited report that exosomal HOTAIR transfers temozolomide resistance through a miR-519a-3p/RRM1 axis in glioblastoma was subsequently the subject of a formal expression of concern (Yuan et al., 2022). Under the present reading, such episodes do not negate the discovery value of EV-HOTAIR studies; rather, they underline why high-impact functional-transfer claims made at the EV or circulation scale require independent technical validation before their language is extended from detection to mechanism. An editorial flag on a single report should not be generalised to the entire circulating-HOTAIR literature, but it does support the need to report detection and demonstrated function separately.

### 6.3 Resistance-monitoring sentinel

Circulating HOTAIR has prospects as a real-time index of resistance—particularly in glioblastoma, where intracellular HOTAIR mechanisms and serum-EV-associated HOTAIR have been linked to temozolomide resistance (Zhang et al., 2020; Lan et al., 2024; Wang X. et al., 2022); circulating lncRNA profiles have also been associated with R-CHOP responsiveness in DLBCL (Senousy et al., 2021).

### 6.4 Intervention: from HOTAIR-targeting drugs to EV network hijacking

Druggable approaches currently sit at D0: EPIC-0628 and AC1Q3QWB disrupt the HOTAIR–EZH2/LSD1 interaction, combined with temozolomide or CDK4/6 inhibitors (Yang et al., 2024; Zhao et al., 2021; Shi et al., 2020). A more ambitious direction inverts the network: since tumours use EVs to disseminate HOTAIR-associated immunosuppressive signals, engineered EVs could in principle be repurposed to deliver HOTAIR-targeting siRNA or ASO back into tumour cells or tumour-supportive immune cells, thereby reframing “disruption” as network hijacking (Alvarez-Erviti et al., 2011; Kamerkar et al., 2022; Mendt et al., 2018; Kamerkar et al., 2017). This remains conceptual specifically for HOTAIR; feasibility depends on resolving the specificity, loading, biodistribution, and dose questions raised in the “Discussion” section.

### 6.5 Translational and regulatory constraints

Beyond cargo selection, EV-based HOTAIR-targeting strategies would face translational and regulatory barriers. Exosome-based therapeutics require reproducible source-cell definition, scalable GMP-compatible manufacturing, identity and purity testing, batch-to-batch consistency, biodistribution analysis, safety assessment, and potency assays. These requirements are particularly important for RNA-loaded EV products because EV heterogeneity and cargo attribution directly affect both mechanism-of-action claims and product classification (Verma and Arora, 2025). A further constraint is that EV tissue access and biodistribution are organ-specific design problems rather than generic properties of exosomes, so delivery to a given target cannot be assumed from EV biology alone (Verma et al., 2025b).

## 7 Discussion

### 7.1 Why the inter-organ horizon remains scientifically attractive

That circulation-scale language sometimes reaches towards inter-organ communication is not mere carelessness—the horizon is biologically plausible. Inter-organ extracellular communication is increasingly documented across systems: adipose-derived circulating exosomal miRNAs can regulate distant tissue gene expression, tumour exosomes can educate bone-marrow progenitors and prepare organ-specific pre-metastatic niches, and microbiota-derived vesicles are increasingly implicated in host and gut–brain signalling (Díaz-Garrido et al., 2021; Thomou et al., 2017; Hoshino et al., 2015; Haas-Neill and Forsythe, 2020). This background plausibility is exactly why occasional over-reading is understandable and why the appropriate response is disciplined reading rather than dismissal. Nevertheless, a modality being possible in principle is not the same as a specific RNA having completed a causal proof; the two should be kept distinct.

### 7.2 The closest attempt, and why it is a D2-exceeding effort rather than inter-organ evidence

The study that comes closest is Lu et al. (2017) on adipose–gut: a sedentary lifestyle, via NF-κB, promotes gluteal-femoral adipose release of exosomal HOTAIR, partially endocytosed by intestinal epithelium and, through Wnt, promoting intestinal stem/progenitor proliferation. Under the distance-aware reading used here, it is best described as a deliberate attempt to exceed circulation-scale inference: it adds *in vivo* causal elements—an adipose-tissue conditional HOTAIR knockout and a compression control in which wild-type but not knockout intestine responds. We discuss it only briefly and do not treat it as established inter-organ evidence because the authors themselves note that selective packaging of HOTAIR into exosomes “…remains to be further identified…”, that intestinal uptake is partial endocytosis rather than demonstrated specific targeting, and that other lncRNAs/miRNAs in the same exosomes may act in the intestine—confounding cargo is not excluded. It is, in short, the most causally complete attempt to move beyond D2, not a demonstration that the inter-organ horizon has been reached.

### 7.3 What it would take to move beyond D2—a falsifiable program

Reaching the horizon requires answering three questions with technologies that now exist. *Specificity*: does donor-organ EV-HOTAIR selectively target a particular recipient organ? Cre-loxP EV-transfer systems fluorescently label recipient cells that have taken up donor EVs *in vivo* (Zomer et al., 2015; Zomer et al., 2016), and Cre-dependent tissue-specific CD63 reporter mice let EVs from a defined tissue be screened and traced to their target (Li et al., 2022; Nørgård et al., 2022; Bettin et al., 2023)—the direct test of the specificity gap noted above (Lu et al., 2017). *Dose–effect*: is circulating HOTAIR, at physiological concentration, sufficient for a measurable recipient-organ phenotype? This needs quantitative single-EV counting plus recipient-organ-specific knockout. *Causal continuity*: can a contiguous donor-to-recipient flow be built rather than stitched correlations? Reporter mice can already close such a flow at the cell-to-cell scale; CD63-GFP mice have traced, *in vivo*, neuronal exosomes carrying a specific miR being taken up by astrocytes and upregulating a glutamate transporter (Men et al., 2019). That proves that a paracrine-scale flow can be technically closed and indicates that the technology for testing the inter-organ horizon exists—not that the horizon has been reached.

### 7.4 What this review is, and is not

**FIGURE 3:**
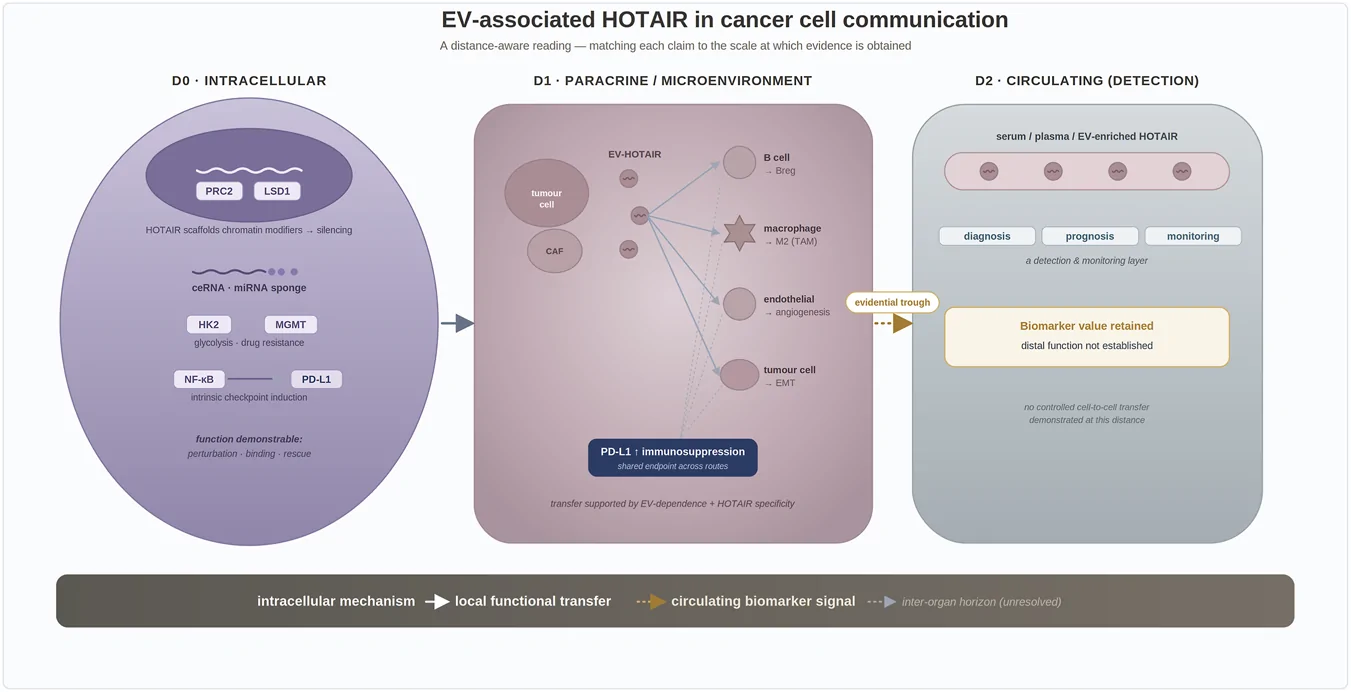
Integrated graphical overview of EV-associated HOTAIR in cancer cell communication. This overview summarises intracellular HOTAIR mechanisms (D0), local EV-associated functional-transfer claims within the tumour microenvironment (D1), and circulating-HOTAIR detection as a biomarker and monitoring layer (D2). The dashed transition from D1 to D2 indicates the evidential trough, where circulating detection is abundant but distal functional communication is not established. The inter-organ horizon is shown as unresolved rather than as an established HOTAIR category.

This is a HOTAIR-centred review, the organising lens of which is offered as a practical interpretative tool for matching cancer cell communication claims to the scale and quality of evidence that supports them. It is not a new doctrine of extracellular-RNA biology and is not a verdict on the circulating-RNA field, which retains clear discovery and monitoring value. A similar “detection exceeding demonstrated function” pattern is visible for other extracellular or circulating lncRNAs, including MALAT1, NEAT1, and H19: these RNAs have been reported as biomarker candidates in plasma, serum, or EV-enriched fractions, yet detection alone does not establish functional extracellular delivery or recipient-cell causality (Badowski et al., 2022; Spanos et al., 2023; Yuan et al., 2020; Zhou et al., 2020). This suggests that the lens may be useful beyond HOTAIR, but we do not claim to have demonstrated that here, because doing so would require a systematic multi-system evaluation outside this review. Our contribution is modest and specific: to separate what circulating-RNA evidence shows from what its language may imply, and to name the gap clearly enough so that it can be tested. An integrated overview of the distance-aware interpretation of EV-associated HOTAIR claims is shown in Figure 3.

### 7.5 Limitations of this review

Several limitations to this review should be acknowledged. As a narrative and concept-driven review, this review was not designed to be systematic or meta-analysis. The cited literature was therefore selected to address the scale-of-evidence question rather than provide a quantitative synthesis of all HOTAIR studies. This design is appropriate for developing a distance-aware interpretative lens, but it also means that the review should not be read as an exhaustive survey of every HOTAIR-related mechanism or cancer type.

A further limitation arises from the uneven structure of the HOTAIR literature itself. Intracellular HOTAIR functions are supported by a relatively broad mechanistic literature, whereas EV-associated or extracellular HOTAIR transfer is supported by a smaller and methodologically heterogeneous set of studies. Many EV-HOTAIR studies rely on *in vitro* co-culture systems, EV-enriched preparations, or correlative circulating-RNA measurements, and these designs do not always establish cytosolic delivery, cargo-specific attribution, donor-specific rescue, or *in vivo* causal continuity. As a result, several conclusions in the field remain sensitive to EV isolation strategy, contamination control, RNA normalisation, and recipient-cell functional assays.

For these reasons, the D0–D1–D2 framework proposed here should be read as a distance-aware interpretative tool for organizing claims and evidentiary gaps, not as a formal grading system or a definitive model of HOTAIR-mediated communication. Its purpose is to make the scale and limits of existing evidence more explicit while identifying the experimental steps required to move from detection or association toward demonstrated functional transfer.

### 7.6 Priority experiments

The principle is claim-matching: as communication claims move farther from the source cell, the required controls should become more explicit, and the wording should remain matched to what the evidence demonstrates. Establishing any ncRNA as a systemic inter-organ messenger should rest on contiguous *in vivo* lineage tracing, not stitched correlation. Priority routes for future cancer-cell-communication studies include tissue-specific EV tagging, single-EV omics, quantitative cargo counting, and conditional EV-secretion knockout. The aim throughout is constructive: to preserve the discovery value of circulating-RNA work while helping its interpretation stay within what it has shown.
